# Differential predictors of early- and delayed-onset post-traumatic stress disorder following physical injury: a two-year longitudinal study

**DOI:** 10.3389/fpsyt.2024.1367661

**Published:** 2024-05-01

**Authors:** Sung-Gil Kang, Ju-Wan Kim, Hee-Ju Kang, Hyunseok Jang, Jung-Chul Kim, Ju-Yeon Lee, Sung-Wan Kim, Il-Seon Shin, Jae-Min Kim

**Affiliations:** ^1^ Department of Psychiatry, Chonnam National University Medical School, Gwangju, Republic of Korea; ^2^ Division of Trauma, Department of Surgery, Chonnam National University Medical School and Hospital, Gwangju, Republic of Korea

**Keywords:** posttraumatic stress disorder, delayed onset, prediction, trauma injury, longitudinal study

## Abstract

**Objectives:**

This study aimed to investigate the predictors of both early- and delayed-onset PTSD over a 2-year period following physical injuries.

**Methods:**

Patients were recruited from a trauma center at a university hospital in South Korea (June 2015 ~ January 2021). At baseline, 1142 patients underwent comprehensive assessments including socio-demographic, pre-trauma, trauma-related, and peri-trauma evaluations. Diagnoses of acute stress disorder (ASD) and subthreshold ASD were also determined using the Clinician-administered PTSD Scale (CAPS). Follow-up assessments at three months included diagnoses of PTSD and subthreshold PTSD using CAPS, and stressful life events (SLEs), with additional evaluations at 6, 12, and 24 months. The analyzed sample comprised 1014 patients followed up at least once after the baseline and 3-month evaluations. PTSD diagnoses were categorized into early-onset (within the first six months after trauma) and delayed-onset (more than six months after trauma). Logistic regression models identified predictors for each group.

**Results:**

Early-onset and delayed-onset PTSD were diagnosed in 79 and 35 patients, respectively. Early-onset PTSD was predicted by previous psychiatric disorders, previous traumatic events, ASD and subthreshold ASD diagnoses, and higher anxiety levels. In contrast, delayed-onset PTSD was linked to higher education, higher injury severity, and subthreshold PTSD and SLEs at 3-month follow-up.

**Conclusion:**

Distinct predictors were found for early-onset and delayed-onset PTSD. The findings underscore the heterogeneous factors influencing the temporal development of PTSD post-trauma, and may provide valuable guidance for more targeted interventions and improved patient outcomes.

## Introduction

The onset of post-traumatic stress disorder (PTSD) often follows significant traumatic experiences, profoundly impacting mental well-being. Identifying predictors of PTSD early on is crucial for prevention and effective management among those exposed to trauma. Numerous potential risk factors have been explored, encompassing socio-demographic variables, pre-trauma health conditions, trauma-related attributes, peri-trauma states, and post-trauma events (1–3).

Despite extensive investigations, findings regarding PTSD predictors have shown significant discrepancies. A systematic review of 44 studies focusing on PTSD predictors in survivors of road traffic accidents revealed inconsistencies, with conflicting outcomes on nearly all variables investigated (4). This variation in findings may be attributed to the complex progression of PTSD, with the majority of cases developing within the first few months but delayed onset occurring beyond six months in 20-30% of cases (5, 6). Many studies have assessed PTSD at specific time points post-incident, ranging from one month to two years (7, 8), potentially mixing both early- and delayed-onset cases.

Studies have examined risk factors, particularly for delayed-onset PTSD. Meta-analyses consistently reported that delayed-onset PTSD often follows previous sub-threshold PTSD (6, 9, 10). However, these analyses suggested that delayed-onset PTSD could occur without previous symptoms, leaving a gap in our understanding. With respect to acute stress disorder (ASD), developed within a month after traumatic events, a previous systematic review of 22 studies suggested that ASD diagnosis alone inadequately identifies the majority of individuals who eventually develop PTSD (11). A meta-regression analysis identified military combat exposure, Western cultural background, and lower cumulative PTSD incidence as associated with delayed-onset PTSD (9). Among patients with physical injuries, factors like initial PTSD symptom severity, mild traumatic brain injury, length of hospitalization, and the number of stressful life events post-injury were identified as risk factors for delayed-onset PTSD (12). In veterans receiving war pensions, delayed-onset PTSD was more frequent in those with absence without leave, disobedience, and dishonesty (13).

To address existing gaps in knowledge, a comprehensive assessment of potential predictors at baseline, along with an exploration of their longitudinal associations with both early- and delayed-onset PTSD, is essential. To our knowledge, there has been no study that investigated the predictors of both early- and delayed-onset PTSD in a study. Utilizing data from a prospective two-year study involving Korean patients with physical injuries, this research aims to contribute valuable insights into the intricate courses of PTSD and the factors influencing them.

## Methods

### Study outline and participants

The present analysis is a part of the Biomarker-based diagnostic algorithm for Post-Traumatic Syndrome (BioPTS) study, which aims to develop precise models for the diagnosis and prediction of PTSD. Detailed information about the study, including its objectives and methodologies, has been previously outlined in a protocol paper (14). To summarize, participants for this study were prospectively recruited from individuals who had recently undergone hospitalization due to physical injuries. The enrollment period spanned from June 2015 to January 2021, and the recruitment occurred at the Department of Trauma Center of Chonnam National University Hospital (CNUH) in Gwangju, South Korea. The severity of injuries sustained by participants was assessed using the Injury Severity Score (ISS) (15) and the Glasgow Coma Scale (GCS) (16). Eligible participants, as detailed in the online supplement, and those expressing willingness to participate, underwent psychiatric assessments within one month of their hospitalization. These assessments were conducted in person. Subsequent follow-up evaluations took place through telephone interviews at intervals of 3, 6, 12, and 24 months following the physical injury event. The final patient visits were completed in January 2023. Ethical clearance for the study was obtained from the Chonnam National University Hospital Institutional Review Board (CNUH 2015-148). To ensure ethical standards, all participants thoroughly reviewed the consent form, and written informed consent was appropriately obtained from each participant.

### Baseline evaluations

#### Socio-demographic characteristics

The following baseline socio-demographic characteristics were documented: age, sex, duration of education, marital status (categorized as currently married or not), cohabitation status (distinguished by living alone or not), religion (noted as religious observance or not), occupational state (categorized as current employed or not), and monthly income (classified as above or below 3,000 USD).

#### Pre-trauma characteristics

Prior histories of psychiatric disorders were documented instances of depressive disorders, panic disorder, agoraphobia, social phobia, and generalized anxiety disorder. Participants’ experiences of previous lifetime traumatic events were examined. Instances of intentional self-harm with some intention to die, irrespective of objective lethality were noted as suicidal attempts (17). Physical disorders were assessed using a questionnaire covering 15 systems or diseases. Personality traits were evaluated using the Big Five Inventory (BFI) (18), and were categorized into resilient and vulnerable type (detailed in online supplement). Childhood abuse experiences were assessed with the Nemesis Childhood Trauma Interview (19), encompassing emotional/psychological, physical, and sexual abuse before the age of 16. A broad definition of “childhood abuse” (having at least one type of abuse) was utilized for the analysis. Resilience and social support were measured by the Connor-Davidson Resilience Scale (CDRS) (20) and the Multidimensional Scale of Perceived Social Support (MSPSS) (21), respectively. Smoking status were categorized into current smoking or not. Alcohol-related problems were screened by the Alcohol Use Disorders Identification Test (AUDIT) (22). Body mass index (BMI) was calculated. Lower CDRS and MSPSS scores and higher AUDIT scores indicate higher symptomatology.

#### Trauma related characteristics

Type of accidental injury was evaluated utilizing the Life Events Checklist for Diagnostic and Statistical Manual of mental disorders, 5th edition (DSM-V) (LEC-5) (23), which aided in identifying the specific type of traumatic event that participants had experienced. To facilitate analysis given the diversity of traumatic events, injury types were categorized into four primary groups: traffic-related injuries, falls (from a height), slips (sideways), and other types of injuries. Injury severity was evaluated with the ISS and GCS as described above, with higher ISS scores and lower GCS scores indicate more pronounced symptomatology. The occurrence of surgical procedures directly related to the injury was documented.

#### Peri-trauma characteristics

During the peri-trauma period spanning from the index injury to the baseline evaluation, participants’ symptoms and functional status were assessed using various evaluation scales. The diagnoses of ASD were determined by the Clinician-Administered PTSD Scale, applying DSM-V criteria (CAPS-5) (24). Diagnosis ASD was established if individuals met nine or more of the 14 symptoms from cluster B. Subthreshold ASD was defined as meeting three of the four symptom clusters (11). Depressive and anxiety symptoms were assessed by the Hospital Anxiety Depression Scale-Depression subscale (HADS-D) and Anxiety subscale (HADS-A) (25), respectively; cognitive function by the Mini-Mental State Examination (MMSE) (26); physical function by the Modified Barthel Index (MBI) (27); subjective cognitive difficulties by the Perceived Deficits Questionnaire-Depression (PDQD) (28); and hypochondriacal fears by the Illness Attitude Scale (IAS) (29). Higher HADS-D, HADS-A, PDQD, and IAS scores; and lower MMSE and MBI scores indicate more severe symptomatology. Physiological status was monitored by measuring vital signs, including systolic and diastolic blood pressures and heart rate, at the time of the baseline evaluation.

### Follow-up evaluations

Following the traumatic events, a structured and systematic follow-up evaluation protocol was implemented at specific intervals to track the development and persistence of post-traumatic stress disorder (PTSD) and subthreshold PTSD, as well as to assess additional relevant factors. The follow-up evaluations were conducted at 3, 6, 12, and 24 months after the traumatic events. At the three months, the CAPS-5 was employed to diagnose PTSD and subthreshold PTSD. PTSD diagnosis required meeting criteria across various symptom clusters: at least one symptom from Cluster B, one from Cluster C, two from Cluster D, one from Cluster E, and meeting Clusters F and G (24). Subthreshold PTSD was defined as having at least one symptom from Cluster B, one symptom from Cluster C, plus 2 symptoms from Cluster D, or one symptom from Cluster B, one symptom from Cluster C, plus 2 symptoms from Cluster E (30). The number of stressful life events (SLEs) experienced during the preceding three months was assessed using the Korean version of the Life Experiences Survey questionnaire (31). Responses were summarized to derive a score, and participants were categorized into groups based on the presence or absence of SLEs. At the subsequent 6, 12, and 24 months follow-up, PTSD diagnoses were reevaluated using the CAPS-5. Follow-up interviews were conducted via telephone, a method validated for its reliability and comparability to face-to-face interviews (32).

### Statistical analysis

Since the delayed onset type of PTSD is defined as PTSD development at least six months after the traumatic events, participants evaluated at least once after both baseline and 3-month evaluations comprised the analyzed sample. This encompassed the evaluation data collected from 3 to 24 months post-traumatic events. PTSD diagnoses were categorized into two groups based on the onset time: early-onset (developed within the first six months after trauma) and delayed-onset (developed more than six months after trauma). Baseline data on socio-demographic characteristics, pre-trauma characteristics, trauma related characteristics, and peri-trauma characteristics, along with 3-month data on subthreshold PTSD and SLEs were compared between absent vs. early-onset group and between absent vs. delayed-onset group using t-tests or χ^2^ tests. Due to limited sample sizes for each group, categorical variables were transformed into binary variables. For example, the four types of accidental injuries were grouped into traffic-related injuries vs. other injuries, considering the distribution of injury types. Variables significantly associated with either early-onset or delayed-onset group (P < 0.05) were entered into separate logistic regression analyses to identify the independent predictors of each group. All statistical tests were two-sided with a significance level set at 0.05. Statistical analyses were carried out using the SPSS 21.0 software.

## Results

### Patient flow and diagnoses of stress-related disorders

**Figure 1:**
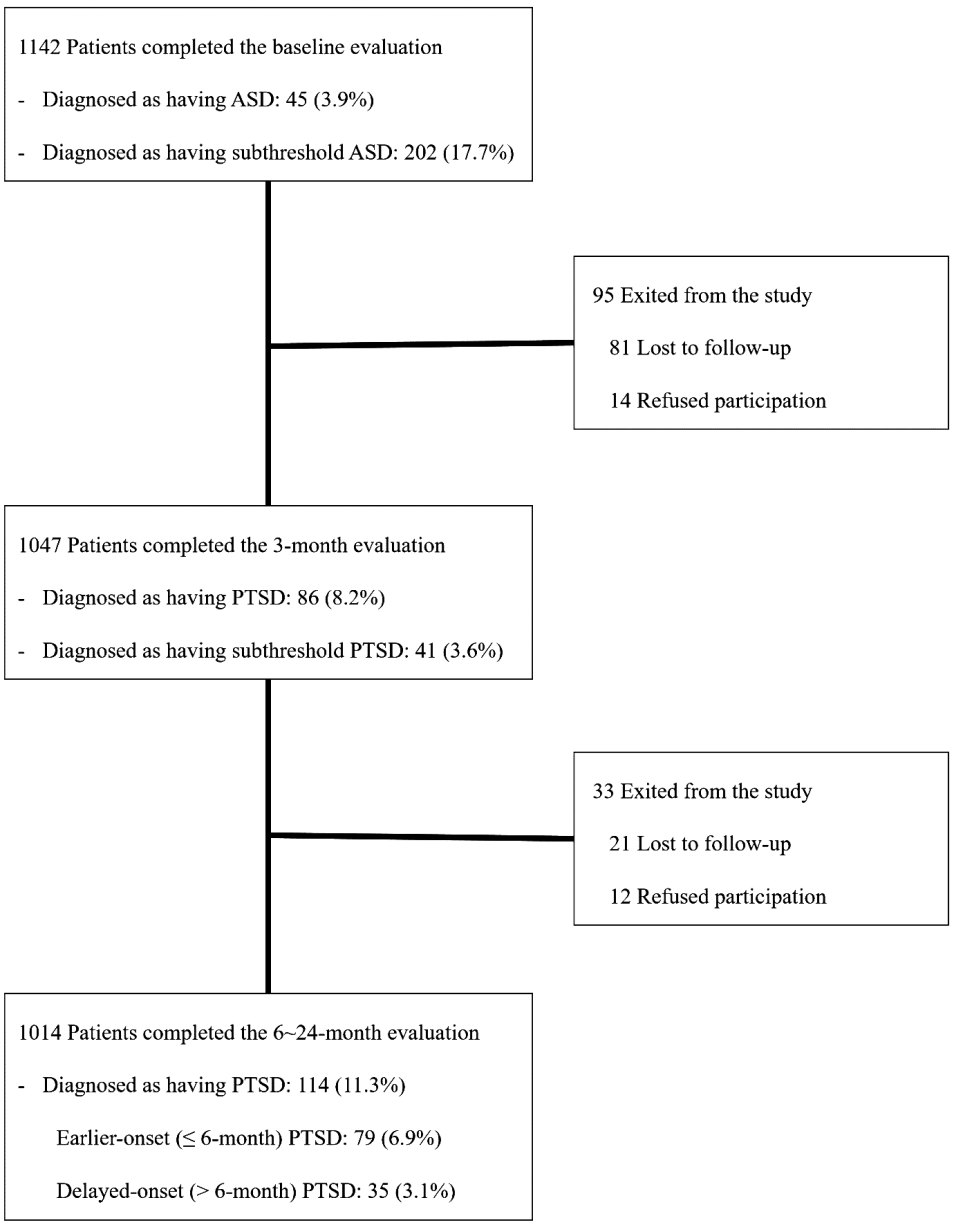
Patient flow by acute stress disorder (ASD) and post-traumatic stress disorder (PTSD) status over 2 years.

The progression of patients with diagnoses of ASD and PTSD from the baseline assessment to the 24-month follow-up is visually depicted in **Figure 1**. Out of 1142 patients met the eligibility criteria and agreed to participate, 45 (3.9%) were diagnosed with ASD, and 202 (17.7%) with subthreshold ASD. At the 3-month follow-up, 1047 (91.7%) of the baseline patients were successfully followed up. Among them, 86 (8.2%) were diagnosed with PTSD, and 41 (3.6%) with subthreshold PTSD. Of the 1142 baseline patients, 1014 (88.8%) were followed up at least once after the baseline and 3-month evaluations, constituting the analyzed sample. Within this sample, early-onset PTSD was diagnosed in 79 individuals (6.9%), and delayed-onset PTSD was diagnosed in 35 individuals (3.1%). No statistical differences were observed in any baseline characteristic between the 1014 patients included in the analyzed sample and the remaining 128 patients (all P-values > 0.1).

### Comparisons by early- and delayed-onset PTSD groups

A detailed comparison between individuals with absent PTSD, those with early-onset PTSD, and those with delayed-onset PTSD is presented in **Table 1**. Early-onset PTSD was significantly associated with lower age, female sex, higher education, religious observance, previous psychiatric history, previous traumatic events, traffic-related injury type, got surgery for the injury, ASD and subthreshold ASD diagnoses, higher scores on HADS-A, HADS-D, MMSE, and IAS. Delayed-onset PTSD was significantly associated with higher education, higher scores on ISS, and subthreshold PTSD and SLEs at 3-month follow-up.

**Table 1:** Baseline characteristics by early- and delayed-onset post-traumatic stress disorder (PTSD) in patients with 1014 physical injuries.

	Absent PTSD (N=900)	Early-onset PTSD (N=79)	Delayed-onset PTSD (N=35)	Absent vs. early-onset PTSD		Absent vs. delayed-onset PTSD	
				Statistical coefficients	P-value^a^	Statistical coefficients	P-value^a^
Socio-demographic characteristics							
Age, mean (SD) years	57.3 (17.3)	51.2 (14.1)	54.6 (14.9)	t=+3.685	**<0.001**	t=+0.925	0.355
Sex, N (%) female	265 (29.4)	38 (48.1)	9 (25.7)	χ^2^=11.829	**0.001**	χ^2^=0.226	0.634
Education, mean (SD) years	10.6 (4.1)	11.8 (3.5)	12.1 (3.4)	t=-2.864	**0.005**	t=-2.595	**0.013**
Marital status, N (%) unmarried	307 (34.1)	22 (27.8)	9 (25.7)	χ^2^=1.277	0.259	χ^2^=1.062	0.303
Living alone, N (%)	142 (15.8)	8 (10.1)	2 (5.7)	χ^2^=1.788	0.181	χ^2^=2.619	0.106
Religious observance, N (%)	361 (40.1)	46 (58.2)	17 (48.6)	χ^2^=9.813	**0.002**	χ^2^=1.001	0.317
Unemployed status, N (%)	164 (18.2)	14 (17.7)	4 (11.4)	χ^2^=0.012	0.912	χ^2^=1.055	0.304
Monthly income, N (%) <3,000 USD	527 (58.6)	38 (48.1)	16 (45.7)	χ^2^=3.252	0.071	χ^2^=2.282	0.131
Pre-trauma characteristics							
Previous psychiatric disorders, N (%)	55 (6.1)	15 (19.0)	3 (8.6)	χ^2^=18.137	**<0.001**	χ^2^=0.350	0.554
Previous traumatic events, N (%)	31 (3.4)	12 (15.2)	3 (8.6)	χ^2^=23.859	**<0.001**	χ^2^=2.527	0.112
Previous suicidal attempt, N (%)	16 (1.8)	3 (3.8)	1 (2.9)	χ^2^=1.557	0.212	χ^2^=0.220	0.639
Number of physical disorders, mean (SD)	2.0 (2.1)	1.5 (1.9)	1.6 (1.8)	t=+1.176	0.076	t=+0.968	0.334
Vulnerable personality, N (%)	392 (43.6)	35 (44.3)	16 (45.7)	χ^2^=0.017	0.898	χ^2^=0.064	0.801
Lower Extraversion, N (%)	504 (56.0)	38 (48.1)	18 (51.4)	χ^2^=1.834	0.176	χ^2^=0.285	0.593
Lower Agreeableness, N (%)	507 (56.3)	47 (59.5)	22 (62.9)	χ^2^=0.295	0.587	χ^2^=0.584	0.445
Lower Conscientiousness, N (%)	387 (43.0)	27 (34.2)	16 (45.7)	χ^2^=2.316	0.128	χ^2^=0.101	0.750
Higher Neuroticism, N (%)	392 (43.6)	35 (44.3)	16 (45.7)	χ^2^=2.129	0.144	χ^2^=0.471	0.493
Lower Openness, N (%)	394 (43.8)	26 (32.9)	15 (42.9)	χ^2^=3.501	0.061	χ^2^=0.012	0.914
Any childhood abuse, N (%)	48 (5.4)	8 (10.1)	4 (11.4)	χ^2^=2.904	0.088	χ^2^=2.256	0.133
Connor-Davidson Resilience Scale, mean (SD) scores	65.2 (15.6)	64.4 (18.1)	68.5 (14.6)	t=+0.413	0.679	t=-1.231	0.219
Multidimensional Scale of Perceived Social Support, mean (SD) scores	34.8 (10.0)	36.1 (11.0)	35.9 (10.1)	t=-1.114	0.265	t=-0.654	0.513
Current smoker, N (%)	240 (26.7)	24 (30.4)	14 (40.0)	χ^2^=0.508	0.476	χ^2^=3.027	0.082
Alcohol Use Disorders Identification Test, mean (SD) scores	10.2 (10.0)	9.3 (10.2)	11.5 (10.0)	t=+0.763	0.446	t=-0.703	0.466
Body mass index, mean (SD)	23.6 (3.4)	23.6 (3.5)	24.3 (3.8)	t=-0.158	0.874	t=-1.203	0.229
Trauma related characteristics							
Injury type, N (%) traffic-related	398 (44.2)	47 (59.5)	17 (48.6)	χ^2^=6.831	**0.009**	χ^2^=0.258	0.611
Injury Severity Score, mean (SD) scores	14.5 (5.8)	14.3 (5.2)	17.1 (7.1)	t=+0.293	0.770	t=-2.560	**0.011**
Glasgow Coma Scale, mean (SD) scores	14.8 (0.7)	14.8 (0.6)	14.9 (0.2)	t=+0.188	0.851	t=-0.830	0.407
Got surgery for the injury, N (%)	462 (51.3)	55 (69.6)	20 (57.1)	χ^2^=9.745	**0.002**	χ^2^=0.455	0.500
Peri-trauma assessment scales and measurements, mean (SD)							
Acute stress disorder (ASD)	21 (2.3)	19 (24.1)	0 (0.0)	χ^2^=87.405	**<0.001**	χ^2^=0.835	0.361
Subthreshold ASD	139 (15.4)	33 (41.8)	8 (22.9)	χ^2^=34.760	**<0.001**	χ^2^=1.397	0.237
Hospital Anxiety Depression scale-anxiety subscale scores	2.7 (3.2)	7.2 (5.1)	2.8 (2.3)	t=-7.668	**<0.001**	t=-0.058	0.954
Hospital Anxiety Depression scale-depression subscale scores	5.2 (4.7)	9.5 (5.4)	6.0 (3.9)	t=-7.011	**<0.001**	t=-1.055	0.292
Mini-Mental State Examination scores	24.3 (5.4)	25.4 (4.3)	23.8 (4.6)	t=-2.077	**0.040**	t=+0.510	0.610
Modified Bathel Index scores	58.2 (31.2)	51.6 (33.4)	55.7 (34.3)	t=+1.802	0.072	t=+0.462	0.644
Perceived Deficits Questionnaire-Depression scores	6.5 (11.5)	7.8 (11.6)	9.1 (13.1)	t=-0.953	0.341	t=-1.294	0.196
Illness Attitude Scale scores	59.3 (20.3)	66.3 (23.1)	55.4 (16.8)	t=-2.586	**0.011**	t=+1.137	0.256
Systolic blood pressure, mmHg	119.7 (13.8)	118.4 (12.2)	115.7 (13.0)	t=+0.769	0.442	t=+1.679	0.094
Diastolic blood pressure, mmHg	72.1 (9.1)	71.3 (9.4)	71.1 (9.0)	t=+0.827	0.408	t=+0.635	0.526
Heart rate per minute	78.3 (11.1)	79.2 (9.9)	77.8 (11.7)	t=-0.720	0.472	t=+0.243	0.808
Characteristics at 3-month, N (%)							
Subthreshold PTSD	34 (3.8)	–	5 (14.3)	–	**-**	χ^2^=9.306	**0.002**
Stressful life events	169 (18.8)	–	22 (62.9)	–	–	χ^2^=40.270	**<0.001**

a analysis of variance or χ^2^ tests, as appropriate. Bold style indicates statistical significance (P-value<0.05).

### Independent predictors of early- and delayed-onset PTSD

**Figure 2:**
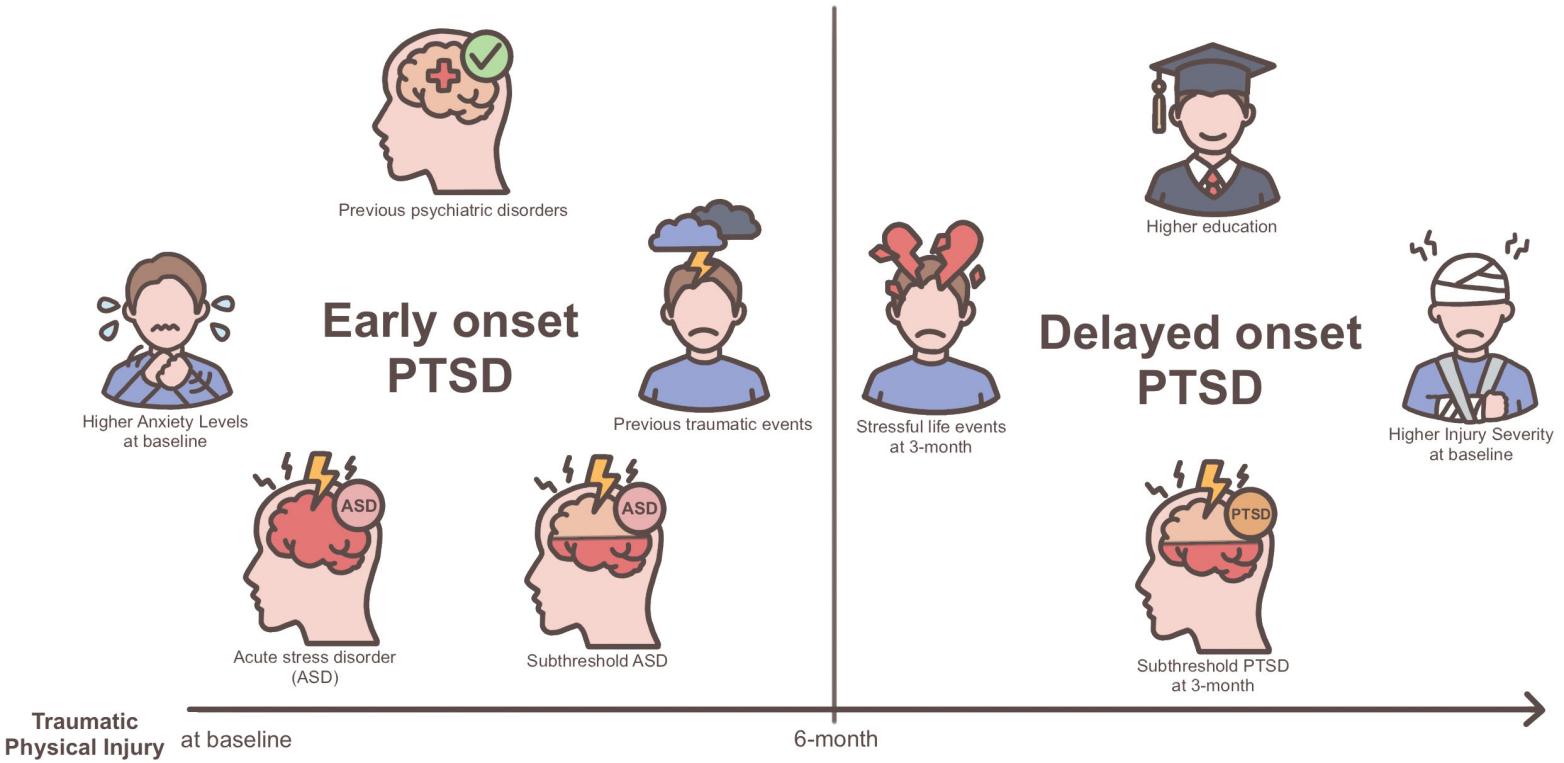
Visualization of predictors for early- vs. delayed-onset post-traumatic stress disorder (PTSD) following physical injury.

Independent predictors of early- and delayed-onset PTSD, identified through logistic regression analyses, are presented in **Tables 2** and **3**, respectively, and are visually summarized in **Figure 2**. Early-onset PTSD was predicted by previous psychiatric disorders, previous traumatic events, ASD and subthreshold ASD diagnoses, and higher scores on HADS-A; and delayed-onset PTSD by higher education, higher scores on ISS, and subthreshold PTSD and SLEs at 3-month follow-up.

**Table 2:** Predictors of early-onset post-traumatic stress disorder in patients with physical injuries.

	OR (95% CI)	P-value
Higher age	0.98 (0.96-1.01)	0.060
Female sex	1.10 (0.61-1.97)	0.762
Higher education	1.06 (0.98-1.16)	0.164
Religious observance	1.49 (0.92-2.42)	0.103
Previous psychiatric disorders	2.20 (1.00-4.45)	**0.049**
Previous traumatic events	3.70 (1.47-9.32)	**0.006**
Traffic-related injury	1.31 (0.75-2.29)	0.340
Got surgery for the injury	1.85 (0.99-3.33)	0.054
Acute stress disorder (ASD)	9.65 (3.70-25.14)	**<0.001**
Subthreshold ASD	4.07 (2.16-7.68)	**<0.001**
Higher Hospital Anxiety Depression scale -anxiety subscale scores	1.10 (1.01-1.20)	**0.038**
Higher Hospital Anxiety Depression scale -depression subscale scores	1.04 (0.97-1.13)	0.273
Higher Mini-Mental State Examination scores	1.03 (0.96-1.11)	0.437
Higher Illness Attitude Scale scores	1.01 (0.99-1.03)	0.071

OR (95% CI): odds ratio (95% confidence interval).

Bold style indicates statistical significance (P-value<0.05).

**Table 3:** Predictors of delayed-onset post-traumatic stress disorder in patients with physical injuries.

	OR (95% CI)	P-value
Higher education	1.10 (1.00-1.21)	**0.049**
Higher Injury Severity Scores	1.05 (1.00-1.11)	**0.048**
Subthreshold PTSD at 3-month	2.86 (1.10-8.60)	**0.036**
Stressful life events at 3-month	6.49 (3.21-13.53)	**<0.001**

OR (95% CI): odds ratio (95% confidence interval).

Bold style indicates statistical significance (P-value<0.05).

## Discussion

This two-year longitudinal study, focusing on patients who experienced physical injuries, revealed distinct predictors for early-onset and delayed-onset PTSD. The findings underscore the heterogeneity in the factors influencing the temporal development of PTSD following traumatic events. Notably, early-onset PTSD was associated with pre-existing vulnerabilities and peri-trauma psychological issues, whereas delayed-onset PTSD was linked to educational levels, trauma severity, and post-trauma characteristics.

The observation that early-onset PTSD is predicted by factors such as previous psychiatric disorders, previous traumatic events, ASD and subthreshold ASD diagnoses, and higher scores on the HADS-A aligns with existing literature. Notably, the association between previous psychiatric disorders and PTSD development within the initial 1-6 months after trauma has been reported in prior studies, reinforcing the consistency of our findings (33, 34). This association can be explained by several factors. Individuals with pre-existing psychiatric disorders may exhibit heightened vulnerability to the impact of trauma, as these conditions can sensitize individuals to stressors (35). Moreover, common risk factors, such as genetic predisposition, childhood adversity, and environmental stressors, may contribute to both pre-existing psychiatric disorders and the development of PTSD (35). Similarly, the association between prior traumatic events and increased vulnerability to PTSD aligns with the sensitization hypothesis. According to this hypothesis, previous exposure to trauma serves as a signal for a greater risk of developing PTSD in response to subsequent traumatic experiences (36). This suggests that the cumulative effect of trauma exposure over time may heighten the individual’s susceptibility to PTSD following new traumatic events.

A prior systematic review encompassing 22 studies suggested that ASD and subthreshold ASD diagnoses exhibited reasonable positive predictive powers but poor sensitivities for predicting the development of PTSD (11). Building upon this knowledge, our study, the first to evaluate both early- and delayed-onset PTSD simultaneously, contributes potentially valuable insights. The results indicate that the predictive values of ASD and subthreshold ASD diagnoses are notably significant for early-onset PTSD but lose significance for delayed-onset PTSD. This suggests that while these diagnoses may robustly predict the immediate emergence of PTSD symptoms, their utility diminishes in predicting delayed onset. Peri-trauma anxiety has been recognized as a predictor of PTSD development both at 1-month (37) and even as far as 3 years following physical injuries (38). The current findings support and extend these earlier studies, emphasizing the impact of peri-trauma anxiety on both early-onset and the enduring effects of PTSD.

Delayed-onset PTSD was predicted by higher education, higher scores on ISS, and the presence of subthreshold PTSD symptoms and SLEs at the 3-month follow-up. Of these factors, subthreshold PTSD symptoms are often present from the traumatic event until diagnosis in the formal diagnosis of delayed-onset PTSD. Our findings are consistent with meta-analyses suggesting that subthreshold PTSD is a common precursor to delayed-onset PTSD (6, 9, 10). The association between SLEs after the index traumatic events and delayed-onset PTSD is in line with previous research in patients with physical injuries (12). Cognitive models suggest that subsequent SLEs may trigger different appraisals of the original traumatic event, contributing to delayed PTSD responses. This emphasizes the ongoing impact of life events in influencing the trajectory of PTSD symptoms over time (39).

The associations between injury severity, particularly higher scores on the ISS, and delayed-onset PTSD can be interpreted in several ways. Firstly, individuals with more severe injuries may initially prioritize survival and physical recovery, delaying the emotional processing of the traumatic experience. This delay may contribute to the onset of PTSD symptoms over time (40). Secondly, individuals with more severe injuries may harbor more negative cognitive appraisals, viewing the trauma as life-threatening or catastrophic. This negative appraisal may contribute to the delayed onset of PTSD symptoms as individuals grapple with the long-term implications of their injuries. Thirdly, individuals with severe injuries may experience ongoing pain, functional disability, and extensive medical interventions, all of which can be traumatic experiences contributing to the delayed onset of PTSD (5).

The unexpected association between higher education and delayed-onset PTSD in our study stands in contrast to most previous research, which typically found either no significant association between education level and PTSD or reported associations with lower education levels. Previous studies have often explained the latter associations by suggesting that individuals with lower education levels may have more limited coping mechanisms when dealing with traumatic events (41, 42). The unexpected nature of our findings could be interpreted in a novel way. It might reflect that individuals with higher levels of education initially cope well shortly after a physical injury, drawing on their cognitive resources and coping strategies. However, over time, they become increasingly aware of potential emotional disturbances linked to their injuries, leading to the delayed onset of PTSD symptoms (43). This perspective suggests that higher education might not be a protective factor in the long term, and individuals with higher education may face unique challenges or processes in the extended aftermath of a traumatic event. These findings underscore the complexity of the relationship between education, psychological factors, and the development of PTSD, and more research is needed to better understand these dynamics.

As highlighted earlier, our study revealed distinctly different risk factors for early- and delayed-onset PTSD. This divergence in risk factors suggests the possibility of different biological bases underlying these two phenotypes, although this particular issue extends beyond the themes addressed in the present study. While previous research has extensively focused on early-onset PTSD, uncovering alterations in neurotransmitter function, hormonal regulation, inflammatory responses, and epigenetic modifications (44, 45), investigations into the biological aspects associated with delayed-onset PTSD are still limited or in an emerging state. The scarcity of research in this domain underscores the need for future studies that delve into neuroimaging, genetics, and molecular biology to gain a more nuanced understanding of the biological underpinnings specific to different PTSD onset patterns.

Strengths of the study include the consecutive recruitment of participants at baseline from all eligible patients who had recently experienced physical injuries, as well as the frequent follow-up assessments reducing the risk of bias arising from heterogeneous examination times. Evaluations and data collection were conducted using a structured research protocol, and well-recognized and standardized scales including the CAPS-5 for PTSD. A broad range of potential risk factors for PTSD were comprehensively investigated at baseline and 3-month follow-up. Long-term follow-up rates were reasonable, and no evidence of selective attrition was found.

Limitations were that this study recruited individuals with physical injuries, limiting the generalizability of findings to those who have experienced other type of traumatic events. However, traumatic physical injuries rank prominently as triggers for PTSD development and are closely linked to enduring impairments in functioning and a diminished quality of life (46). Participants were recruited exclusively from a single trauma center, which may limit the generalizability, although this approach maximized consistency in evaluation and follow-up. The study focused on patients who were hospitalized for moderate to severe physical injuries, and findings cannot necessarily be generalized to individuals with more minor physical injuries. The study did not assess pain levels following injury, both acute and chronic, which have been reported as significant predictors of PTSD development (47). Future research could benefit from incorporating pain assessments to elucidate their role in the development and persistence of PTSD symptoms. Follow-up evaluations were conducted via telephone interview, although this method has proved to be as valid as face-to-face interviews (32).

In conclusion, our study has unveiled distinctive predictors for early- and delayed-onset PTSD over a two-year period following severe physical injuries. These findings underscore the heterogeneity and complexity of PTSD symptom development, emphasizing the need to account for individual characteristics when assessing and addressing PTSD in patients recovering from severe physical injuries. Notably, predicting the onset of delayed PTSD remains challenging due to a lack of comprehensive understanding of its pathogenesis. Our observations contribute to the identification of individuals at risk for delayed-onset PTSD, potentially enabling the development of tailored preventive and management strategies. Recognizing the unique risk factors associated with delayed onset provides valuable insights for clinicians, allowing for more targeted interventions and improved patient outcomes. To enhance the generalizability, future studies should explore multi-center evaluations and include individuals with traumatic events beyond physical injuries. Moreover, further research is imperative to unravel the biological mechanisms underpinning both early- and delayed-onset PTSD.

## Data availability statement

The raw data supporting the conclusions of this article will be made available by the authors, without undue reservation.

## Ethics statement

The studies involving humans were approved by Chonnam National University Hospital Institutional Review Board (CNUH 2015-148). The studies were conducted in accordance with the local legislation and institutional requirements. The participants provided their written informed consent to participate in this study.

## Author contributions

S-GK: Writing – original draft, Writing – review & editing, Investigation. J-WK: Writing – review & editing, Conceptualization, Data curation, Formal analysis, Project administration, Writing – original draft. H-JK: Writing – review & editing, Data curation, Methodology, Validation. J-CK: Writing – review & editing, Data curation, Formal analysis, Methodology. I-SS: Conceptualization, Validation, Writing – review & editing. J-YL: Writing – review & editing. S-WK: Data curation, Project administration, Validation, Writing – review & editing. J-MK: Validation, Writing – review & editing. HJ: Writing – review & editing.
