# Supplementary material for: Differential predictors of early- and delayed-onset post-traumatic stress disorder following physical injury: a two-year longitudinal study
Source: Front Psychiatry. 2024 May 1;15:1367661. doi: 10.3389/fpsyt.2024.1367661 (PMC11094222; doi:10.3389/fpsyt.2024.1367661)
Supplement: Supplementary file 1 [file Presentation_1.pdf]

Online supplementary material

Eligibility criteria

Inclusion criteria were: i) individuals aged 18 years or older at the index injury; ii) patients hospitalised for more than 24 hours after sustaining a moderate to severe physical injury measured by the Injury Severity Score ≥9 (1) ; and iii) individuals sufficiently proficient in the Korean language to comprehend the study protocol. Exclusion criteria were: i) moderate or severe brain injury measured by the Glasgow Coma Scale <10 (2) ; ii) physical injuries resulting from suicide attempts; iii) conditions hindering comprehensive psychiatric evaluation due to severe physical ailments; iv) prior history of psychiatric disorders including psychotic disorder, bipolar disorder, or alcohol or substance use disorders other than depressive and anxiety disorders; v) significant cognitive impairments due to organic mental or neurocognitive disorders; and vi) pre-existing convulsive disorders or a history of anticonvulsant use.

Personality assessments

Personality was assessed by the Big Five Inventory (3). Terminology applied to these five traits has been described as follows: 'Extraversion' - talkative, assertive, and energetic; 'Agreeableness' - good-natured, cooperative, altruistic and empathic; 'Conscientiousness' - orderly, responsible, and dependable; 'Neuroticism' - neurotic, easily upset and not self-confident; and 'Openness' - openness to experience, intellectual, imaginative, and independent-minded. Self-report ratings are made on a scale from 1 to 5 for each item, and higher scores represent higher levels of each given trait. Using the typological approach, personality cluster was identified using cluster analysis. Result of identical analyses with the two-step procedure specified by Asendorpf et al. (4) , two personality types were identified: resilient and vulnerable. Ward's hierarchical clustering procedure was applied for the initial solution; then, iterative k-

means clustering, using Ward's method to define the initial cluster centres, was performed. Each case was assigned to a cluster based on the Euclidean distance from the cluster means; all data was z-standardised to determine the Euclidean distance. The largest shifts in cluster coefficients were observed in the transition from the two- to one-cluster solution; therefore, a two-cluster solution was accepted as the best initial solution. The cluster centres derived from the initial solution were used to implement non-hierarchical k-means clustering.

To evaluate the replicability of the final solutions, the method of Asendorpf et al. (4) was used. Briefly, all cases were randomly split into halves and the full two-step procedure was applied to each half. Next, the participants from each half were assigned to new clusters according to the cluster centres of the other half of the sample. These new clusters were then compared to determine if they agreed with the original clusters according to Cohen's k. A kappa value > 0.60 was required as evidence of replication; the present results satisfied this criterion (replicability coefficients with Cohen's k: 0.833). Compared with the second cluster, the first cluster was characterized by significantly higher extraversion, higher agreeableness, higher conscientiousness, but lower neuroticism (all $p < 0.001$). As defined and labeled in previous studies, the first cluster was labeled as resilient type and the second was vulnerable type (5,6).

References

1. Baker SP, O'Neil B, Haddon W, Long WB. he injury severity score: a method for describing patients with multiple injuries and evaluating emergency care. *J Trauma.* (1974) 14:187-96. doi: 10.1097/00005373-197403000-00001

2. Teasdale G, Jennett B. Assessment of coma and impaired consciousness. A practical scale. *Lancet.* (1974) 2:81-4. doi:10.1016/s0140-6736(74)91639-0

3. John OP, Srivastata S. The Big Five Trait taxonomy: history, measurement, and theoretical

perspertives, in: Pervin, L.A., John, O.P (Eds), Handbook of Personality. 2nd ed. New York: The Guilford Press (1999). 102-38.

4. Asendorpf JB, Borkenau P, Ostendorf F, van Aken MA. Carving personality description at its joints: Confirmation of three replicable personality prototypes for both children and adults. *Eur. J. Pers* (2001) 15:169-98. doi: 10.1002/per.408

5. De Fruyt F. A Person-Centered Approach to P–E Fit Questions Using a Multiple-Trait Model. *J. Vocat. Behav.* (2002) 60:73–90. doi: 10.1006/jvbe.2001.1816

6. Wardenaar KJ, Conradi HJ, Bos EH, de Jonge P. Personality modulates the efficacy of treatment in patients with major depressive disorder. *J Clin. Psychiatry.* (2014) 75:e916-23. doi: 10.4088/JCP.13m08855.
